# Worry about racial discrimination: A missing piece of the puzzle of Black-White disparities in preterm birth?

**DOI:** 10.1371/journal.pone.0186151

**Published:** 2017-10-11

**Authors:** Paula Braveman, Katherine Heck, Susan Egerter, Tyan Parker Dominguez, Christine Rinki, Kristen S. Marchi, Michael Curtis

**Affiliations:** 1 Department of Family and Community Medicine and Center on Social Disparities in Health, University of California, San Francisco, CA, United States of America; 2 School of Social Work, University of Southern California, Los Angeles, CA, United States of America; 3 California Department of Public Health, Maternal Child, and Adolescent Health Division, Sacramento, CA, United States of America; Univesity of Iowa, UNITED STATES

## Abstract

**Objectives:**

The causes of the large and persistent Black-White disparity in preterm birth (PTB) are unknown. It is biologically plausible that chronic stress across a woman’s life course could be a contributor. Prior research suggests that chronic worry about experiencing racial discrimination could affect PTB through neuroendocrine, vascular, or immune mechanisms involved in both responses to stress and the initiation of labor. This study aimed to examine the role of chronic worry about racial discrimination in Black-White disparities in PTB.

**Methods:**

The data source was cross-sectional California statewide-representative surveys of 2,201 Black and 8,122 White, non-Latino, U.S.-born postpartum women with singleton live births during 2011–2014. Chronic worry about racial discrimination (chronic worry) was defined as responses of “very often” or “somewhat often” (vs. “not very often” or “never”) to the question: “Overall during your life until now, how often have you worried that you might be treated or viewed unfairly because of your race or ethnic group?” Prevalence ratios (PRs) with 95% Confidence Intervals (CI) were calculated from sequential logistic regression models, before and after adjustment for multiple social/demographic, behavioral, and medical factors, to estimate the magnitude of: (a) PTB risks associated with chronic worry among Black women and among White women; and (b) Black-White disparities in PTB, before and after adjustment for chronic worry.

**Results:**

Among Black and White women respectively, 36.9 (95% CI 32.9–40.9) % and 5.5 (95% CI 4.5–6.5) % reported chronic worry about racial discrimination; rates were highest among Black women of higher income and education levels. Chronic worry was significantly associated with PTB among Black women before (PR 1.73, 95% CI 1.12–2.67) and after (PR 2.00, 95% CI 1.33–3.01) adjustment for covariates. The unadjusted Black-White disparity in PTB (PR 1.59, 95%CI 1.21–2.09) appeared attenuated and became non-significant after adjustment for chronic worry (PR 1.30, 95% CI 0.93–1.81); it appeared further attenuated after adding the covariates (PR 1.17, 95% CI 0.85–1.63).

**Conclusions:**

Chronic worry about racial discrimination may play an important role in Black-White disparities in PTB and may help explain the puzzling and repeatedly observed greater PTB disparities among more socioeconomically-advantaged women. Although the single measure of experiences of racial discrimination used in this study precluded examination of the role of other experiences of racial discrimination, such as overt incidents, it is likely that our findings reflect an association between one or more experiences of racial discrimination and PTB. Further research should examine a range of experiences of racial discrimination, including not only chronic worry but other psychological and emotional states and both subtle and overt incidents as well. These dramatic results from a large statewide-representative study add to a growing—but not widely known—literature linking racism-related stress with physical health in general, and shed light on the links between racism-related stress and PTB specifically. Without being causally definitive, this study’s findings should stimulate further research and heighten awareness of the potential role of unmeasured social variables, such as diverse experiences of racial discrimination, in racial disparities in health.

## Introduction

Preterm birth (PTB) rates among African-American (“Black”) women have been higher than among European-American (“White”) women in the United States for decades [1, 2]; the causes are poorly understood [1, 3]. For at least two decades, researchers have hypothesized a role for maternal stress in PTB [1–10]. A causal role for stress is biologically plausible based on physiologic mechanisms—including neuroendocrine, vascular, inflammatory, and immune processes—thought to be involved both in stress responses [11] and the initiation of labor [12]. O’Campo, Dunkel Schetter and colleagues [13] have observed higher allostatic load scores among postpartum African American women compared with their White counterparts [13]; allostatic load refers to a set of biomarkers believed to reflect cumulative physiologic wear and tear due to chronic stress and the body’s attempts to adapt [11]. Epigenetic phenomena triggered by chronic stress also have been hypothesized as a contributor to PTB [14, 15]. Chronic stress, even without dramatic events, may be particularly damaging to health [11] it predicts adult chronic disease [11] through mechanisms also thought to be involved in preterm birth [12]. Studies of the association between maternal stress and PTB have produced inconsistent results, however [16–21]. This may reflect the diverse measures of stress and its timing (e.g., lifetime vs during pregnancy and when during pregnancy) used by different studies, along with the complexity of links between stress and PTB, which may be highly dependent on the social and environmental context [6]. While some studies have focused on stress across a woman’s lifetime [6, 16, 22–24], most have focused on stress during pregnancy.

Based on a likely role for stress in PTB, several scholars have hypothesized that experiencing racial discrimination (race-based unfair treatment) may contribute to the Black-White disparity in PTB through physiologic responses to stress [2, 8, 12, 25]. Studies of the relationship between experiences of racial discrimination and the racial disparity in PTB have not been consistent, however. Six of nine published studies on perceived racial discrimination and PTB reported positive associations [5, 26–30] while three did not [31–33]. All of these studies examined the effects of incidents of race-based unfair treatment; none measured worry, anxiety, or the related concept, vigilance (heightened alertness anticipating discriminatory treatment). Vigilance has been linked with many adverse health outcomes [34] but, to our knowledge, has not been studied in relation to birth outcomes.

Experiences of racial discrimination can take multiple forms, including not only overt or subtle discriminatory incidents, but also a range of psychological and emotional reactions to incidents or to awareness of the existence of racism. Even without personally experiencing incidents, individuals may worry or be anxious about potential race-based discrimination based on awareness of pervasive and historical discrimination. People may experience vigilance or concern about confirming negative stereotypes about their social group [35]. They may ruminate about past experiences or experiences of loved ones or of their racial/ethnic group in general, or feel they must perform exceptionally to disprove prejudicial assumptions. Diverse psychological responses to specific incidents or to general awareness of racism, e.g., worry, anger or self-doubt, may occur [8, 34, 36, 37]. We conducted an earlier qualitative study to inform the measurement of experiences of racial discrimination in birth outcomes research [38]. Using semi-structured guides, African-American facilitators asked 40 reproductive-age African-American women in six focus groups to describe their experiences of racial discrimination, racism, or being treated unfairly based on their race. While participants described overt, subtle, and ambiguous incidents, they gave at least as much emphasis to pervasive worry that they or loved ones would experience race-based insults or slights and the need to remain perpetually on guard. Building on those findings and the literature, this study investigated the potential role of chronic worry about race-based unfair treatment (“chronic worry about racial discrimination” or simply “chronic worry”) in the Black-White disparity in PTB, with full awareness that chronic worry about racial discrimination represents only one of many kinds of experiences of racial discrimination that could damage health through pathways involving stress. This study focuses on women’s everyday experiences of racial discrimination in any setting, not specifically on experiences with seeking or receiving health care.

## Methods

### Data source

These analyses used data from the California Maternal and Infant Health Assessment (MIHA) for the years 2011–2014. MIHA is an annual population-based survey of postpartum women in California conducted in English and Spanish since 1999. It is a collaborative effort of the California Department of Public Health (CDPH) and researchers at the University of California, San Francisco (UCSF). The MIHA sample is drawn randomly from statewide birth certificate data each year, stratified by county of birth for large counties and multi-county region for smaller counties, race, and participation in the Special Supplemental Nutrition Program for Women Infants and Children (WIC). African-American women are oversampled to obtain sufficient numbers for analyses. Mothers under age 15, those with quadruplet or higher-order births, and those with out-of-state or missing addresses are excluded from the sample. Surveys are mailed to sampled women two months after delivery, and non-respondents are sent a reminder postcard and a second survey; remaining non-respondents are followed-up by telephone. Most surveys are completed by 4 months postpartum. Approximately two-thirds of the surveys are completed by mail and one-third by telephone using Computer Assisted Telephone Interviewing (CATI). Survey responses are linked to each woman’s information from her baby’s birth certificate. MIHA’s annual response rates during 2011–2014 were 70–71% overall, 63–71% among Black women, and 69–72% among White women, depending on the year. Overall, 6,810 to 7,010 women participated each year during 2011–2014, for a total sample during the study period of 27,626.

The MIHA study is conducted in accordance with prevailing ethical principles. The protocol and instruments for each year were approved by the Institutional Review Boards of the California Health and Human Services Agency and the University of California, San Francisco. The MIHA data set includes sensitive information that could potentially identify individual participants who have delivered a baby. To ensure confidentiality, all data are kept on password-protected, encrypted servers to which only MIHA team personnel have access. In accordance with strict procedures to prevent release of such information, CDPH policy prohibits making MIHA data publicly available. Although CDPH is working with its legal team to update MIHA’s protocol to allow dissemination of a limited de-identified dataset for research purposes, procedures are not currently in place to permit such dissemination. Current protocols only allow members of the MIHA team at CDPH and UCSF to access and analyze the data.

### Sample and weighting

From the 27,626 MIHA participants during 2011–2014, this study selected 2,201 Black and 8,122 White non-Latino women with singleton births who had gestational age information and responded to the MIHA question about how often they worried about discrimination. Immigrants were excluded because their experiences of racial discrimination might differ from those of U.S.-born women [39]. Only singleton births were included because PTB causes may differ for multiple births [40, 41].

MIHA data are weighted to be representative of women giving birth in California during the index year who meet the inclusion criteria. Weighting includes components reflecting the sampling fraction based on the sampling frame and all eligible births in each county or multi-county region each year; nonresponse probabilities; and iterative adjustments using raking to make weights reflect the population of eligible women giving birth in each large county or multi-county sampling region. (S1 Table shows the characteristics and PTB rates of unweighted MIHA respondents, non-respondents, the final weighted sample, and all eligible women giving birth statewide during 2011–2014, based on birth certificate information on U.S.-born non-Latino Black and White women. Respondents were a little older, more likely to be White and not to have a PTB compared with nonrespondents; respondents were also more highly educated, more likely to have private insurance and less likely to have Medi-Cal (California’s Medicaid program) coverage. The final weighted sample was similar to the statewide target population.)

### Variables

Unless stated otherwise, variables were based on self-reported information in MIHA.

*The outcome variable*: Based on birth certificate information on gestational age and National Center for Health Statistics criteria, *PTB* was defined as birth between 17 and 36 completed weeks of gestation [42]. The obstetric estimate was used for all but 10 births for which gestational age was calculated from last menstrual period because the obstetric estimate was missing.

*The exposure variable* was *chronic worry about race-based unfair treatment (or racial discrimination)*, also referred to here as “chronic worry,” defined by responses to the question: “Overall during your life until now, how often have you worried that you might be treated or judged unfairly because of your race or ethnic group?” The responses “very often” or “somewhat often” (in contrast to “not very often” or “never”) were categorized as chronic worry (worrying often) about racial discrimination.

*Racial group* was classified as Black or White based on birth certificate data, using the first-listed group when multiple groups were reported.

*The following covariates*, based on self-reported information in MIHA unless otherwise noted, were considered potential confounders of associations among chronic worry about racial discrimination, PTB, and racial group. We selected covariates that at least theoretically could be related both to (a) PTB and either (b) chronic worry about racial discrimination or (c) racial group, recognizing that some of the covariates might mediate the relationships studied, producing conservative estimates.

*The social/demographic covariates* included maternal demographic, socioeconomic, and psychosocial factors. The demographic factors were age and parity (from birth certificates), and marital status. The socioeconomic factors were family income as a percent of Federal Poverty Guidelines, educational attainment, and percent of persons in a respondent’s residential census-tract with household incomes below poverty (geocoded from birth certificate addresses; with tract-level poverty rates obtained from the American Community Survey). The psychosocial factors during pregnancy were depressive symptoms [43] and number (0, 1, 2–3, 4+) of major stressors during pregnancy; the stressors were: food insecurity (using the 6-item USDA measure) [44]; having to move due to problems paying the rent or mortgage; homelessness or no regular place to sleep; separation or divorce; a serious drug or alcohol problem of someone close to the respondent; intimate partner violence [43]; and the respondent’s or her partner’s involuntary job loss, reduced pay or hours, or incarceration.

*The behavioral covariates* reflected known or widely suspected risk factors for PTB: smoking in the 3 months before pregnancy (likely reflecting fetal exposure early in pregnancy and more complete reporting than smoking during pregnancy) [http://www.cdc.gov/prams/pdf/phase7_core-questions.pdf]; binge drinking (4 or more drinks at one sitting) during pregnancy; and unintended pregnancy (unwanted, mistimed, or unsure) [http://www.cdc.gov/prams/pdf/phase-7-core-questions-508.pdf].

*The medical covariates* reflected medical or medical-care risk factors: inter-pregnancy interval (based on birth certificate data on dates of prior birth and last menstrual period); self-reported pre-pregnancy overall health status; a diagnosis by a health care provider of diabetes or high blood pressure/hypertension before this pregnancy; first-trimester prenatal care initiation; pre-pregnancy underweight (body mass index <18.5 kg/m2); and inadequate gestational weight gain (adjusted for gestational duration, using birth certificate and MIHA data and Institute of Medicine (now National Academy of Medicine) guidelines) [45].

Most covariates had data missing for less than 1% of both Black and White women. The three covariates with the highest missing rates were family income (6.4% of Black and 3.5% of White women), census-tract poverty (5.6% of Black and 3.6% of White women), and number of stressors (2.1% of Black and 1.0% of White women). Missing values for family income and census-tract poverty were imputed using hot-deck imputation with several socioeconomic and behavioral predictors; although virtually unchanged, results are reported using imputed data. After imputation, 6.1% of women were still missing one or more variables and were excluded from fully adjusted models, giving a final sample size of 9,698 for these models. Rates of chronic worry about racial discrimination and rates of PTB (overall and among those who did and did not report chronic worry) were similar whether including or excluding records with missing data (shown in S2 Table).

### Analyses

*Analyses* were performed using SAS version 9.4 [http://www.sas.com/en_us/software/sas9.html] and SUDAAN version 11 [https://www.rti.org/impact/sudaan-statistical-software-analyzing-correlated-data]. Survey data analysis procedures were used to adjust for MIHA’s complex sampling design.

*Descriptive analyses*. Distributions of covariates and prevalence of reporting chronic worry about racial discrimination by each covariate were examined separately among Black and White women, as were PTB rates overall and PTB rates by reports of chronic worry.

*Analyses examining associations between chronic worry about racial discrimination and PTB*, *among Black women and White women examined separately*. While chronic worry about discrimination among Black women was of primary interest, we also studied White women, given our interest in the Black-White disparity in PTB. To examine the unadjusted association between chronic worry about race-based unfair treatment and PTB separately among Black and White women, prevalence ratios (PR) were calculated from predicted marginal probabilities of logistic regression models comparing PTB prevalence among women who did and did not report chronic worry. We initially adjusted only for chronic worry to assess its potential impact without other covariates, next adding the social/demographic variables, and finally behavioral and medical variables that are often assumed to be involved in PTB disparities by race.

*Analyses examining the role of chronic worry about racial discrimination in the Black-White disparity in PTB*. Similarly, PRs were calculated investigating PTB rates among Black women compared with White women, examining how adjustment for chronic worry and the covariates (added sequentially as in the models examining Black women and White women separately, described above) affected the Black-White difference in PTB prevalence.

## Results

Table 1 describes Black and White women’s characteristics and how the prevalence of chronic worry about racial discrimination varied with those characteristics. Over one-third (36.9%, 95% CI 32.9–40.9%) of Black women and 5.5% (CI 4.5–6.5%) of White women reported chronic worry. Among Black (but not White) women, those who were older, married, and had higher income or education levels were more likely to report chronic worry. For White women, lower levels of income and education were associated with reporting chronic worry. Most medical variables were not associated with chronic worry, but among Black women, those who reported being diagnosed with hypertension before pregnancy were more likely to report chronic worry.

**Table 1:** Self-reported characteristics, and prevalence of chronic worry about racial discrimination (chronic worry) by those characteristics, among eligible U.S.-born non-Latino Black and White women with singleton live births in California, MIHA 2011–2014.

	U.S.-born Black women (n = 2,201)		U.S.-born White women (n = 8,122)	
Characteristics	% distribution of characteristics Column %	% who reported chronic worry* Row %	% distribution of characteristics Column %	% who reported chronic worry* Row %
Overall	100.0	36.9 (32.9–40.9)	100.0	5.5 (4.5–6.5)
***Social/demographic factors***				
Maternal age		ⱡ		ⱡ
15–19	10.6 (7.8–13.4)	24.7 (15.1–34.3)	3.1 (2.5–3.6)	8.6 (3.0–14.2)
20–24	30.7 (26.9–34.6)	30.5 (24.2–36.8)	15.3 (13.9–16.7)	9.7 (6.2–13.1)
25–29	26.5 (22.4–30.5)	40.0 (31.2–48.7)	28.8 (26.9–30.7)	5.0 (3.2–6.7)
30–34	21.3 (17.4–25.1)	44.7 (34.8–54.6)	32.6 (30.7–34.5)	4.6 (3.1–6.2)
35+	10.9 (8.4–13.5)	44.2 (32.5–55.9)	20.2 (18.4–22.0)	4.0 (1.8–6.1)
Parity		ⱡ		
Primiparous	42.0 (37.5–46.4)	33.3 (27.0–39.6)	45.5 (43.4–47.6)	5.0 (3.3–6.6)
2–3 births	41.2 (37.2–45.1)	42.8 (37.0–48.6)	47.8 (45.7–49.9)	5.9 (4.6–7.3)
4+ births	16.9 (13.2–20.6)	31.2 (21.5–40.9)	6.7 (5.8–7.6)	5.9 (3.2–8.6)
Marital status		ⱡ		
Married	26.0 (22.4–29.6)	47.5 (39.4–55.6)	73.1 (71.4–74.8)	5.0 (3.8–6.2)
Living with a partner	29.5 (25.3–33.7)	34.6 (26.7–42.6)	16.6 (15.2–18.0)	6.5 (4.6–8.4)
Single, separated, divorced, widowed	44.5 (40.2–48.8)	31.9 (26.7–37.1)	10.3 (9.0–11.5)	7.7 (3.8–11.6)
Family income		ⱡ		ⱡ
< = 100% poverty	65.6 (62.0–69.3)	31.8 (27.0–36.7)	21.3 (19.8–22.9)	9.0 (6.5–11.4)
101–200%	18.9 (15.6–22.2)	44.3 (34.8–53.8)	15.9 (14.4–17.4)	7.3 (5.0–9.7)
>200%	15.5 (13.2–17.8)	49.7 (40.8–58.5)	62.8 (60.9–64.7)	3.8 (2.6–5.1)
Education		ⱡ		ⱡ
Less than high school	13.8 (11.1–16.5)	33.8 (24.1–43.4)	5.1 (4.2–5.9)	9.7 (5.5–13.8)
High school/GED	24.2 (20.4–28.1)	24.7 (18.1–31.2)	11.6 (10.4–12.8)	6.6 (4.0–9.1)
Some college	45.3 (41.0–49.6)	40.5 (34.3–46.7)	37.6 (35.6–39.6)	6.2 (4.5–8.0)
College graduate	16.7 (13.5–19.8)	47.5 (36.7–58.3)	45.7 (43.7–47.8)	4.1 (2.6–5.5)
Percent of census-tract residents with incomes below poverty level				ⱡ
<5%	4.5 (3.3–5.7)	33.8 (21.5–46.1)	18.4 (16.7–20.1)	2.7 (1.4–4.0)
5-<10%	11.3 (8.8–13.7)	38.0 (26.9–49.0)	29.5 (27.6–31.4)	5.3 (3.4–7.2)
10-<20%	29.2 (25.0–33.4)	39.4 (31.0–47.8)	33.3 (31.3–35.2)	6.2 (4.3–8.1)
>20%	55.0 (50.7–59.4)	35.6 (30.4–40.8)	18.9 (17.3–20.4)	7.2 (4.7–9.6)
Number of major stressors during pregnancy		ⱡ		ⱡ
0	43.4 (38.9–48.0)	29.1 (23.4–34.9)	65.3 (63.3–67.2)	3.7 (2.6–4.8)
1	23.4 (19.8–27.1)	38.9 (29.9–48.0)	18.1 (16.5–19.7)	5.8 (3.7–7.9)
2–3	23.3 (19.6–27.0)	47.4 (38.6–56.2)	12.4 (11.1–13.7)	9.9 (6.8–13.0)
4 or more	9.8 (7.4–12.3)	47.6 (35.5–59.7)	4.2 (3.4–5.0)	18.0 (8.9–27.1)
Depressive symptoms during pregnancy		ⱡ		ⱡ
Yes	21.1 (17.5–24.8)	48.2 (38.7–57.7)	9.9 (8.8–11.1)	10.7 (7.6–13.8)
No	78.9 (75.2–82.5)	33.9 (29.3–38.5)	90.1 (88.9–91.2)	4.9 (3.9–6.0)
***Behavioral factors***				
Smoked in the 3 months pre-pregnancy				ⱡ
Yes	20.4 (16.8–24.0)	41.2 (32.1–50.3)	19.6 (18.0–21.2)	7.9 (5.3–10.5)
No	79.6 (76.0–83.2)	35.1 (30.8–39.4)	80.4 (78.8–82.0)	4.9 (3.9–6.0)
Binge drank during pregnancy		ⱡ		
Yes	6.1 (4.3–7.8)	55.1 (41.0–69.3)	6.8 (5.8–7.8)	7.3 (1.6–12.9)
No	93.9 (92.2–95.7)	35.2 (31.3–39.1)	93.2 (92.2–94.2)	5.4 (4.4–6.4)
Unintended pregnancy				ⱡ
Yes ^a^	66.2 (62.0–70.5)	35.5 (31.0–40.0)	33.4 (31.4–35.3)	7.4 (5.4–9.4)
No ^b^	33.8 (29.5–38.0)	39.3 (31.9–46.8)	66.6 (64.7–68.6)	4.3 (3.3–5.4)
***Medical/medical care factors***				
Lacked first-trimester prenatal care		ⱡ		ⱡ
Yes	16.7 (13.2–20.2)	28.6 (20.6–36.5)	7.7 (6.7–8.7)	8.3 (5.6–11.1)
No	83.3 (79.8–86.8)	38.6 (34.1–43.1)	92.3 (91.3–93.3)	5.3 (4.2–6.3)
Interpregnancy interval (multiparous women)				
<6 months	6.1 (3.5–8.6)	31.9 (16.0–47.9)	3.7 (2.5–4.9)	3.4 (0.0–6.9)
6–11 months	12.9 (9.3–16.6)	32.6 (20.1–45.1)	12.2 (10.5–13.9)	5.8 (3.0–8.7)
12–23 months	16.8 (12.3–21.2)	38.3 (25.5–51.1)	32.5 (30.0–35.1)	5.1 (3.3–6.8)
24+ months	64.2 (58.7–69.8)	41.8 (35.0–48.6)	51.6 (48.9–54.3)	6.7 (4.7–8.7)
Self-reported health pre-pregnancy				ⱡ
Poor or fair	9.8 (7.5–12.1)	40.6 (29.6–51.6)	3.8 (3.2–4.5)	11.7 (6.8–16.7)
Good, very good, or excellent	90.2 (87.9–92.5)	36.4 (32.1–40.7)	96.2 (95.5–96.8)	5.3 (4.2–6.3)
Diabetes diagnosis pre-pregnancy				
Yes	2.2 (1.3–3.1)	27.8 (10.3–45.2)	1.2 (0.7–1.6)	6.3 (0.9–11.7)
No	97.8 (96.9–98.7)	37.1 (33.0–41.2)	98.8 (98.4–99.3)	5.3 (4.4–6.3)
Hypertension diagnosis pre-pregnancy		ⱡ		
Yes	6.2 (4.3–8.1)	56.4 (41.2–71.7)	2.8 (2.1–3.5)	3.9 (1.3–6.6)
No	93.8 (91.9–95.7)	35.6 (31.5–39.7)	97.2 (96.5–97.9)	5.6 (4.5–6.6)
Underweight (BMI<18.5) pre-pregnancy				
Yes	4.7 (3.1–6.3)	29.8 (13.1–46.4)	4.3 (3.3–5.3)	3.5 (0.5–6.4)
No	95.3 (93.7–96.9)	37.1 (32.9–41.2)	95.7 (94.7–96.7)	5.6 (4.5–6.6)
Pregnancy weight gain				
Inadequate	19.5 (16.0–22.9)	37.5 (28.6–46.3)	14.7 (13.1–16.2)	4.2 (2.6–5.8)
Adequate or excessive	80.5 (77.1–84.0)	36.5 (31.9–41.1)	85.3 (83.8–86.9)	5.7 (4.6–6.9)

* Chronic worry about racial discrimination (“chronic worry”) was defined as responding “very often” or”somewhat often” to the question: “Overall during your life until now, how often have you worried that you might be treated or judged unfairly because of your race or ethnic group?”

ⱡ Chi-square test for difference in percentage reporting chronic worry across the categories (within a racial group) was significant.

^a^ Unwanted, mistimed, or unsure

^b^ Wanted to get pregnant then

Table 2 displays numbers and rates of PTB among Black women and White women overall and according to whether they reported chronic worry. Overall, 9.2% (CI 7.2–11.2%) of Black and 5.8% (CI 4.8–6.8%) of White women had a PTB. Among Black women, PTB occurred among 12.5% (CI 8.4–16.7%) of those reporting chronic worry and 7.2% (CI 5.3–9.2%) who did not. Among White women, 9.9% (CI 2.6–17.1%) of those reporting chronic worry about racial discrimination had a PTB, compared with 5.6% (CI 4.6–6.5%) of those who did not. (S3 Table includes the information in Table 2 and displays below it the numbers and rates of PTBs according to each of the social/demographic, behavioral, and medical/medical care covariates. PTB rates appeared to vary by many characteristics, but confidence intervals generally overlapped. Patterns by age, income, and education tended to mirror observations in the literature, with apparently higher rates of PTB among Black women (but not among White women) with higher levels of income. The most striking differences in PTB rates by characteristics were seen for binge drinking among White women, and for diagnoses of diabetes or hypertension before pregnancy among Black women.)

**Table 2:** Prevalence of preterm births overall by whether women reported chronic worry about racial discrimination (chronic worry) among U.S.-born non-Latino Black and White women with singleton live births in California, MIHA 2011–2014.

	U.S-born Black women (n = 2,201)		U.S.-born White women (n = 8,122)	
	N Preterm	% Preterm (95%CI)	N Preterm	% Preterm (95%CI)
**Overall preterm birth rate**	234	9.2 (7.2–11.2)	492	5.8 (4.8–6.8)
Reported chronic worry	105	12.5 (8.4–16.7)	30	9.9 (2.6–17.1)
Did not report chronic worry	129	7.2 (5.3–9.2)	462	5.6 (4.6–6.5)

Note: S3 Table includes the information in Table 2 and in addition displays preterm birth prevalence associated with each of the covariates used in this study.

Separately for Black women and for White women, Table 3 shows prevalence ratios (PR) and 95% Confidence Intervals (CI) for the association between chronic worry about racial discrimination and PTB, first unadjusted, then adjusted for each cluster of covariates, and then adjusted for all covariates. Without adjustment, the PR comparing PTB rates among Black women reporting chronic worry with those who did not was 1.73 (CI 1.12–2.67); the PR among Black women was similar after adjustment for the social/demographic covariates alone (1.95, CI 1.27–2.97) and all covariates (PR 2.00, CI 1.33–3.01). Corresponding estimates for White women appeared similar but slightly lower than PRs among Black women after adjustment; none was statistically significant. (S4 Table shows the results in Table 3 and in addition for all variables in the model corresponding to Table 3. On examining the PRs for the association between each covariate and PTB adjusting for all other covariates, the only variables that were significantly associated with PTB among Black women were neighborhood poverty (with greater neighborhood poverty rates paradoxically protective), fair or poor prepregnancy health, hypertension before pregnancy, and inadequate weight gain. For White women the only significant variables were parity, marital status, neighborhood poverty, binge drinking, and diabetes before pregnancy.)

**Table 3:** Prevalence ratios for preterm birth associated with chronic worry about racial discrimination among U.S.-born non-Latino Black and White women with singleton live births in California, MIHA 2011–2014.

Variables included in model	Prevalence ratio (and 95% CI) for PTB among women who reported chronic worry about racial discrimination relative to those who did not	
	U.S.-born Black women (n = 2,201)	U.S.-born White women (n = 8,122)
Chronic worry about racial discrimination (unadjusted)	1.73 (1.12–2.67)	1.77 (0.83–3.77)
Chronic worry about racial discrimination and social/demographic covariates*	1.95 (1.27–2.97)	1.67 (0.73–3.79)
Chronic worry about racial discrimination and social/demographic*, behavioral**, and medical*** covariates	2.00 (1.33–3.01)	1.84 (0.91–3.71)

*Social/demographic covariates: maternal age, parity, marital status, family income, maternal education, % of census-tract residents with incomes below poverty guideline, number of major stressors during pregnancy, depressive symptoms during pregnancy

**Behavioral covariates: smoked in 3 months before pregnancy, binge drank while pregnant, unintended pregnancy

***Medical covariates: lacked first-trimester prenatal care, interpregnancy interval, self-reported health pre-pregnancy, diabetes was diagnosed before pregnancy, hypertension was diagnosed before pregnancy, underweight pre-pregnancy, inadequate (vs adequate or excessive) pregnancy weight gain

Note: S4 Table includes the information in Table 3 and in addition displays the prevalence ratios associated with each of the covariates.

Table 4 displays results of analyses examining the Black-White disparity in PTB. The unadjusted PR for PTB rates among Black women compared with White women was 1.59 (CI 1.21–2.09). After adjusting for chronic worry about racial discrimination, the Black-White PTB difference appeared attenuated and was no longer statistically significant (PR 1.30, CI 0.93–1.81); it was 1.08 (CI 0.76–1.54) after adding the social/demographic variables, then 1.17 (CI 0.85–1.63) after adding the behavioral and medical variables. (S5 Table includes the information in Table 4 and in addition displays the PRs for each covariate. S6 Table shows that adding only the behavioral and medical variables without the social/demographic variables to the model produced negligible changes in the PR adjusted only for chronic worry.)

**Table 4:** Prevalence ratios comparing PTB prevalence among U.S.-born non-Latino Black relative to White women with singleton live births in California, before and after adjustment for chronic worry about racial discrimination and covariates, MIHA 2011–2014.

Variables included in model	Prevalence ratio (and 95% CI) for PTB among Black relative to White women
Racial group (unadjusted)	1.59 (1.21–2.09)
Racial group and chronic worry about racial discrimination	1.30 (0.93–1.81)
Racial group and chronic worry about racial discrimination and social/demographic covariates*	1.08 (0.76–1.54)
Racial group and chronic worry about racial discrimination and social/demographic*, behavioral**, and medical*** covariates	1.17 (0.85–1.63)

*Social/demographic covariates: maternal age, parity, marital status, family income, maternal education, % of census-tract residents with incomes below poverty guideline, number of major stressors during pregnancy, depressive symptoms during pregnancy

**Behavioral covariates: smoked in 3 months before pregnancy, binge drank while pregnant, unintended pregnancy

***Medical covariates: lacked first-trimester prenatal care, interpregnancy interval, self-reported health pre-pregnancy, diabetes was diagnosed before pregnancy, hypertension was diagnosed before pregnancy, underweight pre-pregnancy, inadequate (vs adequate or excessive) pregnancy weight gain

Note: S5 Table includes the data in Table 4 and in addition displays the prevalence ratios associated with each of the covariates.

## Discussion

Results of this large, statewide-representative study suggest that chronic worry about racial discrimination may play an important role in the Black-White disparity in PTB. Although this study cannot establish a causal role for chronic worry in the elevated risk of PTB among Black women, a causal role is plausible based on current understanding of the physiologic mechanisms through which chronic stress could contribute to premature birth [12]. These results add to growing evidence linking adverse health outcomes with a range of experiences of racial discrimination, including diverse psychological and emotional states as well as incidents [34, 46, 47]. The results add to literature linking racial discrimination specifically with PTB [5, 26–30] by suggesting a possible link between PTB risk and a psychological state—namely, chronic worry about the possibility of being treated unfairly based on one’s race.

Among the more than one in three Black women who reported chronic worry about race-based unfair treatment, the prevalence of PTB was approximately twice that among Black women who did not report chronic worry. This difference persisted even after adjustment for many other factors that would tend to diminish the observed associations with chronic worry. If chronic worry about racial discrimination does contribute causally to increased rates of PTB among Black women, the potential impacts—in terms of suffering, unfulfilled human potential because of the developmental sequellae of PTB, and economic costs for society—could be staggering.

A limitation of this study is the fact that our measure of chronic worry about racial discrimination has not been formally tested psychometrically. It was, however, systematically developed based on qualitative research [38] (see Introduction) and pretested cognitively and in focus groups. Another limitation is that we did not have information on the extent to which women worried about other issues, apart from racial discrimination. Our finding, however, that Black women, as expected, reported chronic worry about racial discrimination far more frequently than White women supports the likelihood that this measure captures worry about racial discrimination rather than more generalized anxiety or worry. Furthermore, the observed patterns of association between our measure of chronic worry and other variables in this study (e.g., education and income) are consistent with previous findings linking experiences of discrimination with depressive symptoms [34, 37] smoking [48] and other unhealthy behaviors [37]. In addition, the higher prevalence of chronic worry about racial discrimination observed here among more socioeconomically advantaged Black women is consistent with findings from previous research on perceived incidents of discrimination [34].

Another key limitation is that while our data source included only one question on experiences of discrimination, i.e., worry about racial discrimination, racism-related experiences can encompass diverse experiences [38], as reflected by multi-component scales [36, 49–51]. One cannot be certain whether worry about racial discrimination, as opposed to other unmeasured racism-related psychological states or incidents that are associated with worry, was responsible for the associations observed in this study. It is highly likely, however, that the findings reflect a link between PTB and one or more experiences of racism.

Another potential limitation is that given the retrospective nature of this study, the observed association between chronic worry about racial discrimination and PTB may reflect recall bias. If, however, having experienced PTB made women subsequently more likely to recall or report prior adverse experiences (such as worry about racial discrimination at any time in the past), one might expect to observe associations between PTB and women’s reports of other adverse events or circumstances such as depressive symptoms and major stressors during pregnancy. In this study, we found no association between PTB and depressive symptoms among Black or White women. Of the nine major stressors that were examined, furthermore, none was associated with PTB among Black women, and only two were associated with PTB among White women (shown in S7 Table).

Our findings also suggest that White women who report chronic worry about racial discrimination may be at elevated risk of PTB. One can only speculate about what the U.S.-born non-Latino White women in this study meant when they reported worrying about being treated or judged unfairly based on their race or ethnic group. Did they experience racial tensions or social exclusion in their neighborhoods or workplaces, or resent what they perceived as others’ more favorable treatment? These are not unlikely possibilities given that reporting of chronic worry about racial discrimination was more prevalent among White women of lower educational and income levels. Although experiences of racial discrimination are likely to be different for Black and White women, we believe it is valid not only to examine potential effects of worry about racial discrimination among Black women but also to assess its potential contributions to the Black-White disparity in PTB, which requires including White women in analyses. This analytic approach is supported by previous literature [8, 28, 48, 51].

We considered the possible role of multiple racial identifications among women whose first-listed race was White. Overall, 3.2% of White women reported a second or third non-White race; for less than 1% (n = 71), Black/African-American was listed as a second or third race (data on maternal and paternal race among White women. Preterm birth rates did not differ between White women who also reported Black or other races and those who reported only White race. (S8 Table) We also considered whether White (as first-listed race) women whose babies had fathers who were Black or of other racial/ethnic groups, may have worried more frequently about discrimination because of their inter-racial relationships. Based on birth certificate data on paternal as well as maternal race/ethnicity, the 281 out of 8,122 White (as first-listed race) women with partners whose first, second, or third race was listed as Black, had higher rates of chronic worry about racial discrimination than White women with White partners; however, these women did not have higher rates of preterm birth than did women with non-Latino White partners.(shown in S8 Table)

The socioeconomic variation observed in the prevalence of chronic worry about race-based unfair treatment may shed light on a perplexing finding from some earlier studies. While PTB rates among White women generally decrease with more income or education, this has not been observed consistently among Black women [52–56], suggesting that Black women have not always reaped the expected health benefits (e.g., improved birth outcomes) of greater socioeconomic resources. This is consistent with the findings of other researchers [57–59] who have observed that African Americans appeared to derive less health benefit from higher levels of educational attainment than Whites. Our findings of both higher rates of chronic worry about racial discrimination among more socioeconomically-advantaged Black women and an association between chronic worry about racial discrimination and PTB may help explain this apparent paradox. It is conceivable that Black women of higher income or education levels could worry more about racial discrimination than their less socioeconomically-advantaged Black counterparts because they may interact more frequently with Whites; they may be more likely to be in the minority in their workplaces and potentially where they shop, study, reside, and obtain services [60]. They may feel–and may have felt for a long time—that they constantly need to prove themselves against negative stereotypes. Black women of higher socioeconomic status may therefore paradoxically experience more chronic racism-related stress. Cole and Omari [61] discussed the “hidden costs of upward mobility” for middle-class African Americans, including encountering glass ceilings to advancement at work, potential alienation from other African Americans, and facing frequent demands for financial support from less well-off family and friends, all of which could be stressful.

While this study was not designed to reveal the underlying mechanisms for the relationship between chronic worry about racial discrimination and higher risks of PTB among Black women, our findings indicate that this relationship was not explained by the health-related behaviors or medical factors we measured. These findings are limited, however, by concerns about the reliability of self-reports of medical diagnoses. It is possible that unmeasured differences in factors such as economic or social hardships during childhood, stressful experiences before pregnancy, neighborhood characteristics apart from poverty rates, accumulated wealth, or unmeasured aspects of any covariate may be important.

The persistence in some studies of a Black-White disparity in PTB after adjustment for socioeconomic variables has sometimes been interpreted by default to reflect underlying biological differences. Our findings, however—that the magnitude and statistical significance of Black-White differences in PTB prevalence appeared markedly attenuated after adjusting for chronic worry about racial discrimination, with and without other covariates—suggest an important role for social factors, many of which are rarely measured. There is a growing consensus among researchers that the causes of PTB are likely to be complex and multifactorial, and this study’s findings do not challenge that assumption; they do, however, indicate a potentially important role in the Black-White disparity in PTB for Black women’s experiences of racial discrimination. While neither causally definitive nor indicative of an easy medical or policy “fix,” our results add to mounting evidence of the likely toll of racism-related stress on important physical health outcomes in general [46, 47, 62] and specifically suggest a potentially major role for racism-related stress in the racial disparity in PTB, which should be further studied. The findings underscore the need, when studying health disparities by racial or ethnic group, to consider unmeasured social differences, including differences in potentially powerful chronic stressors such as experiences of racial discrimination. Given the difficulty of measuring all relevant social variables in any research, this study highlights the need for awareness that underlying biological differences should not be a default explanation of racial differences in health.

## Supporting information

S1 Table Characteristics of respondents, non-respondents, final weighted sample, and target population. (PDF)

S2 Table PTB and chronic worry rates including and excluding records with missing covariates. (PDF)

S3 Table PTB rates by chronic worry and by each covariate, separately among Black and White women. (PDF)

S4 Table Prevalence ratios for PTB associated with chronic worry & each covariate, separately among Black & White women. (PDF)

S5 Table Prevalence ratios for PTB associated with race and for each covariate. (PDF)

S6 Table Prevalence ratios for PTB associated with race, adjusting only for chronic worry and behavioral/medical covariates. (PDF)

S7 Table Significance of associations between maternal stressors during pregnancy and PTB. (PDF)

S8 Table PTB and chronic worry rates according to detailed maternal and paternal race. (PDF)
