# Supplementary material for: Worry about racial discrimination: A missing piece of the puzzle of Black-White disparities in preterm birth?
Source: PLoS One. 2017 Oct 11;12(10):e0186151. doi: 10.1371/journal.pone.0186151 (PMC5636124; doi:10.1371/journal.pone.0186151)
Supplement: S1 Table — (PDF) [file pone.0186151.s001.pdf]

S1 Table. Comparison of characteristics and PTB rates of U.S.-born non-Latino White and Black respondents and non-respondents to the MIHA survey, the final weighted sample, and the statewide target population of all eligible women giving birth in California, 2011-2014. Sources: Birth certificate data for U.S.-born non-Latino Black and White women.

	Respondents (unweighted) (n=10,732)	Non-respondents (n=4,421)	Weighted sample (n=10,732)	Target population (n=589,511)
Maternal age				
15-19	5.3	7.1	4.3	4.6
20-24	19.1	23.2	17.8	18.1
25-29	28.1	29.7	28.1	27.6
30-34	30.0	24.7	30.6	30.3
35 or older	17.5	15.4	19.3	19.5
Maternal education				
Less than high school graduate	7.5	12.2	6.1	6.7
High school graduate/GED	23.0	30.7	21.7	22.4
Some college	33.1	34.4	32.4	31.7
College graduate	36.4	22.8	39.8	39.2
Delivery payer				
Medi-Cal	38.7	48.0	28.9	29.2
Private insurance	55.0	44.5	63.7	62.5
Military	1.3	1.7	1.3	1.3
Other	1.8	2.2	4.3	5.1
Uninsured/Self-pay	3.1	3.6	1.9	1.9
WIC program participation				
Received WIC during pregnancy	33.3	29.8	32.5	33.9
Did not receive WIC	66.7	70.2	67.5	66.1
Preterm birth				
Yes	8.1	9.2	7.5	7.6
No	91.9	90.8	92.5	92.4
Race/ethnicity				
White, non-Latino	78.6	74.4	83.0	82.9
Black, non-Latino	21.4	25.6	17.0	17.1
