# Supplementary material for: Worry about racial discrimination: A missing piece of the puzzle of Black-White disparities in preterm birth?
Source: PLoS One. 2017 Oct 11;12(10):e0186151. doi: 10.1371/journal.pone.0186151 (PMC5636124; doi:10.1371/journal.pone.0186151)
Supplement: S2 Table — (PDF) [file pone.0186151.s002.pdf]

S2 Table. Rates of chronic worry about racial discrimination and preterm birth, including and excluding records with any missing covariates, U.S.-born non-Latino Black and White women with singleton births in California, MIHA 2011-2014.

	U.S.-born Black women		U.S.-born White women	
	All women (n=2,201)	Excluding those with any missing covariates (n=1,986)	All women (n=8,122)	Excluding those with any missing covariates (n=7,712)
Preterm birth rate	9.2 (7.2-11.2)	9.6 (7.4-11.7)	5.8 (4.8-6.8)	5.4 (4.5-6.4)
Among women reporting chronic worry	12.5 (8.4-16.7)	13.8 (9.1-18.5)	9.9 (2.6-17.1)	9.5 (1.9-17.1)
Among women not reporting chronic worry	7.2 (5.3-9.2)	7.1 (5.0-9.2)	7.2 (4.6-6.5)	5.2 (4.3-6.1)
Rate of worry about racial discrimination	36.9 (32.9-40.9)	36.2 (32.2-40.3)	5.5 (4.5-6.5)	5.2 (4.3-6.1)
