# Supplementary material for: Worry about racial discrimination: A missing piece of the puzzle of Black-White disparities in preterm birth?
Source: PLoS One. 2017 Oct 11;12(10):e0186151. doi: 10.1371/journal.pone.0186151 (PMC5636124; doi:10.1371/journal.pone.0186151)
Supplement: S3 Table — (PDF) [file pone.0186151.s003.pdf]

S3 Table. Prevalence of preterm births overall, by whether women reported chronic worry about racial discrimination (chronic worry), and by each covariate, among U.S.-born non-Latino Black and White women with singleton live births in California, MIHA 2011-2014.

	U.S-born Black women (n=2,201)		U.S.-born White women (n=8,122)	
	N Preterm	% Preterm (95%CI)	N Preterm	% Preterm (95%CI)
**Overall preterm birth rate**	234	9.2 (7.2-11.2)	492	5.8 (4.8-6.8)
Reported chronic worry	105	12.5 (8.4-16.7)	30	9.9 (2.6-17.1)
Did not report chronic worry	129	7.2 (5.3-9.2)	462	5.6 (4.6-6.5)
**Covariates**				
Social/demographic factors				
Maternal age				
15-19	23	9.3 (2.4-16.2)	20	5.2 (2.1-8.3)
20-24	55	7.6 (4.6-10.6)	91	7.0 (3.9-10.0)
25-29	51	5.9 (3.6-8.3)	137	5.2 (3.9-6.5)
30-34	71	15.0 (8.7-21.3)	132	4.6 (3.1-6.0)
35+	34	10.3 (4.6-16.1)	112	7.8 (4.9-10.8)
Parity				
Primiparous	81	7.1 (4.9-9.3)	248	7.2 (5.4-9.0)
2-3 births	102	10.1 (6.9-13.3)	194	4.1 (3.1-5.1)
4+ births	51	12.4 (5.6-19.3)	50	8.7 (5.2-12.3)
Marital status				
Married	60	9.1 (5.5-12.7)	301	5.1 (4.0-6.2)
Living with a partner	73	10.0 (5.4-14.6)	118	7.1 (5.0-9.1)
Single, separated, divorced, widowed	99	8.8 (6.2-11.4)	72	8.8 (4.3-13.3)
Family income				
<=100% poverty	142	7.9 (5.9-10.0)	145	6.6 (4.7-8.5)
101-200%	45	11.5 (5.2-17.8)	95	6.6 (3.7-9.5)
>200%	43	11.6 (6.2-16.9)	250	5.3 (4.1-6.6)
Education				
Less than high school	39	10.3 (5.5-15.0)	32	4.2 (2.0-6.4)
High school/GED	57	9.7 (5.5-13.9)	85	8.8 (4.8-12.7)
Some college	98	8.1 (5.0-11.1)	201	6.3 (4.7-7.9)
College graduate	40	10.7 (5.5-15.8)	174	4.8 (3.5-6.1)
Percent of census-tract residents with incomes below poverty level				

<5%	21	21.3 (9.2-33.3)	60	4.2 (2.1-6.3)
5-<10%	27	13.4 (3.7-23.1)	141	5.9 (4.1-7.8)
10-<20%	71	7.3 (4.7-9.9)	163	6.0 (4.3-7.8)
>20%	113	8.3 (5.9-10.7)	126	6.8 (4.6-9.0)
Number of major stressors during pregnancy				
0	83	8.6 (5.5-11.6)	291	5.6 (4.4-6.8)
1	67	11.6 (6.1-17.1)	96	5.4 (3.5-7.4)
2-3	53	7.7 (4.7-10.7)	66	6.8 (3.2-10.4)
4 or more	25	9.8 (4.3-15.3)	34	5.9 (3.3-8.5)
Depressive symptoms during pregnancy				
Yes	65	7.6 (4.6-10.5)	73	6.2 (3.9-8.5)
No	168	9.6 (7.2-12.0)	418	5.8 (4.7-6.8)
Behavioral factors				
Smoked in the 3 months pre-pregnancy				
Yes	63	10.1 (6.2-14.0)	130	7.5 (4.7-10.2)
No	171	9.2 (6.8-11.5)	360	5.4 (4.4-6.5)
Binge drank during pregnancy				
Yes	11	9.8 (1.3-18.3)	39	13.1 (6.3-19.9)
No	219	9.3 (7.2-11.4)	449	5.3 (4.3-6.2)
Unintended pregnancy				
Yes [a]	141	8.8 (6.2-11.4)	204	6.5 (4.8-8.2)
No [b]	91	10.0 (6.8-13.2)	284	5.2 (4.0-6.3)
Medical/medical care factors				
Lacked first-trimester prenatal care				
Yes	43	11.4 (6.1-16.8)	67	8.8 (4.9-12.7)
No	191	8.8 (6.6-10.9)	425	5.5 (4.5-6.6)
Interpregnancy interval (multiparous women)				
<6 months	5	12.5 (0.0-27.8)	12	5.2 (1.0-9.4)
6-11 months	20	12.7 (3.0-22.4)	28	3.9 (1.8-6.0)
12-23 months	24	7.2 (2.9-11.6)	62	4.0 (2.1-5.8)
24+ months	101	11.1 (7.2-15.0)	139	5.2 (3.8-6.6)
Self-reported health pre-pregnancy				

Poor or fair	42	14.9 (7.9-22.0)	39	13.1 (6.4-19.7)
Good, very good, or excellent	191	8.6 (6.5-10.6)	453	5.5 (4.5-6.5)
Diabetes diagnosis pre-pregnancy				
Yes	22	29.2 (12.9-45.5)	17	15.7 (5.6-25.7)
No	206	8.8 (6.7-10.8)	470	5.7 (4.7-6.7)
Hypertension diagnosis pre-pregnancy				
Yes	42	28.3 (12.8-43.7)	31	8.4 (3.9-13.0)
No	188	8.0 (6.2-9.9)	455	5.7 (4.7-6.7)
Underweight (BMI<18.5) pre-pregnancy				
Yes	10	11.7 (2.4-21.0)	23	7.0 (2.3-11.7)
No	223	9.1 (7.1-11.2)	468	5.8 (4.7-6.8)
Pregnancy weight gain				
Inadequate	62	14.3 (8.5-20.2)	92	5.9 (3.8-8.0)
Adequate or excessive	170	8.0 (6.0-10.1)	397	5.6 (4.6-6.6)

*Chronic worry about racial discrimination ("chronic worry") was defined as responding "very often" or "somewhat often" to the question: "Overall during your life until now, how often have you worried that you might be treated or judged unfairly because of your race or ethnic group."

Note: This table includes the information in Table 2 and in addition displays the prevalence of preterm birth by each covariate.
