# Supplementary material for: Worry about racial discrimination: A missing piece of the puzzle of Black-White disparities in preterm birth?
Source: PLoS One. 2017 Oct 11;12(10):e0186151. doi: 10.1371/journal.pone.0186151 (PMC5636124; doi:10.1371/journal.pone.0186151)
Supplement: S4 Table — (PDF) [file pone.0186151.s004.pdf]

S4 Table. Prevalence ratios for preterm birth associated with chronic worry about racial discrimination among U.S.-born Black women and White women with singleton live births in California, MIHA 2011-2014.

Variables included in model	Prevalence ratio (and 95% CI) for PTB among women who reported chronic worry about racial discrimination relative to those who did not	
	U.S.-born Black women (n=2,201)	U.S.-born White women (n=8,122)
Chronic worry about racial discrimination (unadjusted)	1.73 (1.12-2.67)	1.77 (0.83-3.77)
Chronic worry about racial discrimination, adjusted for social and demographic covariates	1.95 (1.27-2.97)	1.67 (0.73-3.79)
Social/demographic covariates:		
Age (ref: 35+)		
15-19	1.35 (0.43-4.22)	0.37 (0.14-0.98)
20-24	1.04 (0.47-2.28)	0.59 (0.28-1.27)
25-34	1.03 (0.55-1.93)	0.59 (0.39-0.89)
Parity (ref: 2-3 births)		
Primiparous	0.71 (0.39-1.29)	1.79 (1.22-2.61)
4+ births	1.59 (0.94-2.68)	1.92 (1.19-3.10)
Marital status (ref: Married)		
Living with a partner	1.47 (0.84-2.59)	1.42 (0.98-2.08)
Single, separated, divorced, widowed	1.48 (0.86-2.53)	1.85 (1.05-3.26)
Family income (ref: 200% poverty+)		
<=100% poverty	0.68 (0.38-1.21)	0.92 (0.54-1.57)
101-200% poverty	1.05 (0.57-1.95)	0.98 (0.62-1.54)
Education (ref: College graduate)		
Less than high school graduate	1.14 (0.54-2.42)	0.83 (0.41-1.70)
High school graduate/GED	1.13 (0.59-2.14)	1.62 (0.85-3.07)
Some college	0.78 (0.43-1.4)	1.33 (0.89-1.98)
Neighborhood poverty (ref: <5%)		
5-9.9%	0.71 (0.32-1.59)	1.33 (0.75-2.38)
10-19.9%	0.37 (0.18-0.77)	1.23 (0.71-2.11)
>=20%	0.49 (0.26-0.93)	1.44 (0.78-2.67)
Number of stressors (ref: 0)		
1	1.27 (0.78-2.07)	0.83 (0.55-1.25)
2-3	0.89 (0.52-1.51)	0.91 (0.55-1.51)
4 or more	1.08 (0.51-2.30)	0.63 (0.34-1.19)
Depression during pregnancy (ref: no)		
Yes	0.66 (0.40-1.09)	1.03 (0.65-1.63)
Chronic worry about racial discrimination and social/demographic, behavioral, and medical covariates	2.00 (1.33-3.01)	1.84 (0.91-3.71)

Social/demographic covariates:		
Age (ref: 35+)		
15-19	1.38 (0.53-3.59)	0.38 (0.14-1.03)
20-24	1.02 (0.48-2.20)	0.61 (0.30-1.22)
25-34	0.97 (0.52-1.80)	0.56 (0.37-0.86)
Parity (ref: 2-3 births)		
Primiparous	0.76 (0.43-1.34)	1.69 (1.14-2.52)
4+ births	1.44 (0.85-2.43)	1.77 (1.10-2.85)
Marital status (ref: Married)		
Living with a partner	1.44 (0.89-2.33)	1.56 (1.08-2.26)
Single, separated, divorced, widowed	1.27 (0.75-2.13)	1.96 (1.10-3.48)
Family income (ref: 200% poverty+)		
<=100% poverty	0.79 (0.42-1.49)	0.79 (0.48-1.31)
101-200% poverty	1.18 (0.61-2.28)	1.02 (0.66-1.59)
Education (ref: College graduate)		
Less than high school graduate	0.98 (0.45-2.15)	0.80 (0.38-1.66)
High school graduate/GED	1.07 (0.54-2.11)	1.53 (0.80-2.92)
Some college	0.80 (0.44-1.47)	1.33 (0.91-1.94)
Neighborhood poverty (ref: <5%)		
5-9.9%	0.65 (0.32-1.33)	1.77 (1.13-2.79)
10-19.9%	0.38 (0.20-0.71)	1.55 (1.01-2.39)
>=20%	0.48 (0.27-0.83)	1.73 (0.99-3.00)
Number of stressors (ref: 0)		
1	1.04 (0.65-1.67)	0.88 (0.59-1.31)
2-3	0.81 (0.47-1.40)	0.88 (0.53-1.47)
4 or more	0.88 (0.39-2.02)	0.79 (0.46-1.36)
Depression during pregnancy (ref: no)		
Yes	0.62 (0.35-1.09)	0.95 (0.62-1.45)
Behavioral covariates:		
Smoking in 3 months before pregnancy (ref: no)		
Yes	1.08 (0.62-1.88)	0.95 (0.64-1.41)
Binge drinking during pregnancy (ref: no)		
Yes	1.07 (0.49-2.38)	2.10 (1.21-3.64)
Unintended pregnancy (ref: no)		
Yes	0.76 (0.48-1.19)	0.83 (0.58-1.19)
Medical covariates:		
Lacked first-trimester prenatal care (ref: no)		
Yes	1.23 (0.73-2.09)	1.31 (0.81-2.11)
Interpregnancy interval (ref: 24+ months or primiparous)		
<6 months	1.03 (0.34-3.07)	1.28 (0.56-2.90)
6-11 months	1.28 (0.61-2.67)	0.95 (0.53-1.72)
12-23 months	0.66 (0.32-1.35)	1.01 (0.60-1.69)
Self-reported health pre-pregnancy (ref: good, very good, or excellent)		
Fair or poor	1.75 (1.05-2.91)	1.47 (0.89-2.41)
Diabetes diagnosis pre-pregnancy (ref:		

no)		
Yes	2.04 (0.95-4.40)	2.47 (1.13-5.41)
Hypertension diagnosis pre-pregnancy (ref: no)		
Yes	2.54 (1.42-4.53)	1.01 (0.45-2.22)
Underweight (BMI<18.5) pre-pregnancy (ref: no, BMI ≥18.5)		
Yes	1.67 (0.80-3.48)	1.31 (0.64-2.66)
Inadequate weight gain (ref: adequate or excessive)		
Yes	1.64 (1.06-2.54)	0.95 (0.65-1.37)

Note: This table includes the information in Table 3 and in addition displays the prevalence ratios associated with each covariate in the models.
