# Supplementary material for: Worry about racial discrimination: A missing piece of the puzzle of Black-White disparities in preterm birth?
Source: PLoS One. 2017 Oct 11;12(10):e0186151. doi: 10.1371/journal.pone.0186151 (PMC5636124; doi:10.1371/journal.pone.0186151)
Supplement: S5 Table — (PDF) [file pone.0186151.s005.pdf]

S5 Table. Prevalence ratios comparing PTB prevalence among U.S.-born non-Latino Black relative to White women with singleton live births in California, before and after adjustment for chronic worry about racial discrimination and covariates, MIHA 2011-2014.

Variables included in model	Prevalence ratio (and 95% CI) for PTB among Black relative to White women
Racial group: U.S.-born Black (unadjusted); ref=U.S.-born White	1.59 (1.21-2.09)
Racial group (adjusted for chronic worry about racial discrimination)	1.30 (0.93-1.81)
Chronic worry about racial discrimination	1.76 (1.13-2.73)
Racial group (adjusted for chronic worry about racial discrimination and social/demographic covariates)	1.08 (0.76-1.54)
Chronic worry about racial discrimination	1.83 (1.17-2.88)
Social/demographic covariates:	
Age (ref: 35+)	
15-19	0.51 (0.22-1.17)
20-24	0.62 (0.32-1.18)
25-34	0.63 (0.43-0.92)
Parity (ref: 2-3 births)	
Primiparous	1.48 (1.05-2.07)
4+ births	1.83 (1.23-2.74)
Marital status (ref: Married)	
Living with a partner	1.49 (1.07-2.08)
Single, separated, divorced, widowed	1.70 (1.09-2.66)
Family income (ref: 200% poverty+)	
<=100% poverty	0.80 (0.52-1.24)
101-200% poverty	1.02 (0.69-1.51)
Education (ref: College graduate)	
Less than high school graduate	1.16 (0.68-2.00)
High school graduate/GED	1.57 (0.93-2.67)
Some college	1.23 (0.86-1.76)
Neighborhood poverty (ref: <5%)	
5-9.9%	1.26 (0.76-2.08)
10-19.9%	1.04 (0.64-1.68)
>=20%	1.20 (0.71-2.02)
Number of stressors (ref: 0)	
1	0.96 (0.69-1.34)
2-3	0.93 (0.61-1.42)
4 or more	0.79 (0.49-1.28)
Depression during pregnancy (ref: no)	
Yes	0.88 (0.61-1.26)

Racial group (adjusted for social/demographic, behavioral, and medical covariates)	1.17 (0.85-1.63)
Chronic worry about racial discrimination	1.98 (1.31-2.99)
Social/demographic covariates:	
Age (ref: 35+)	
15-19	0.55 (0.24-1.26)
20-24	0.65 (0.35-1.19)
25-34	0.62 (0.42-0.92)
Parity (ref: 2-3 births)	
Primiparous	1.39 (0.98-1.97)
4+ births	1.71 (1.16-2.52)
Marital status (ref: Married)	
Living with a partner	1.62 (1.18-2.23)
Single, separated, divorced, widowed	1.70 (1.06-2.71)
Family income (ref: 200% poverty+)	
<=100% poverty	0.74 (0.49-1.11)
101-200% poverty	1.06 (0.72-1.56)
Education (ref: College graduate)	
Less than high school graduate	1.07 (0.60-1.91)
High school graduate/GED	1.49 (0.87-2.57)
Some college	1.22 (0.87-1.72)
Neighborhood poverty (ref: <5%)	
5-9.9%	1.55 (1.03-2.33)
10-19.9%	1.25 (0.85-1.85)
>=20%	1.34 (0.84-2.13)
Number of stressors (ref: 0)	
1	0.98 (0.71-1.36)
2-3	0.90 (0.59-1.39)
4 or more	0.81 (0.51-1.30)
Depression during pregnancy (ref: no)	
Yes	0.81 (0.58-1.15)
Behavioral covariates:	
Smoking in 3 months before pregnancy (ref: no)	
Yes	0.99 (0.71-1.38)
Binge drinking during pregnancy (ref: no)	
Yes	1.90 (1.16-3.12)
Unintended pregnancy (ref: no)	
Yes	0.83 (0.61-1.12)
Medical covariates:	
Lacked first-trimester prenatal care (ref: no)	
Yes	1.23 (0.85-1.79)
Interpregnancy interval (ref: 24+ months or primiparous)	
<6 months	1.30 (0.63-2.71)
6-11 months	1.06 (0.65-1.74)
12-23 months	0.88 (0.57-1.38)
Self-reported health pre-pregnancy (ref:	

good, very good, or excellent)	
Fair or poor	1.55 (1.06-2.26)
Diabetes diagnosis pre-pregnancy (ref: no)	
Yes	2.27 (1.25-4.11)
Hypertension diagnosis pre-pregnancy (ref: no)	
Yes	1.63 (0.95-2.78)
Underweight (BMI<18.5) pre-pregnancy (ref: no, BMI $\geq$18.5)	
Yes	1.40 (0.81-2.43)
Inadequate weight gain (ref: adequate or excessive)	
Yes	1.17 (0.87-1.59)

Note: This table includes the information in Table 4 and in addition displays the prevalence ratio for preterm birth among Black women relative to White women, by each covariate.
