# Supplementary material for: Worry about racial discrimination: A missing piece of the puzzle of Black-White disparities in preterm birth?
Source: PLoS One. 2017 Oct 11;12(10):e0186151. doi: 10.1371/journal.pone.0186151 (PMC5636124; doi:10.1371/journal.pone.0186151)
Supplement: S6 Table — (PDF) [file pone.0186151.s006.pdf]

S6 Table. Prevalence ratios comparing PTB prevalence among U.S.-born non-Latino Black relative to White women with singleton live births in California, before and after adjustment for chronic worry about racial discrimination, showing the results in Table 4 along with results associated with adjusting only for chronic worry and the behavioral/medical variables, MIHA 2011-2014.

Variables included in model	Prevalence ratio (and 95% CI) for PTB among Black relative to White women
Racial group: U.S.-born Black (unadjusted); ref=U.S.-born White	1.59 (1.21-2.09)
Racial group (adjusted for chronic worry about racial discrimination)	1.30 (0.93-1.81)
Racial group (adjusted for chronic worry about racial discrimination and behavioral/medical covariates)	1.29 (0.94-1.75)
Racial group (adjusted for chronic worry about racial discrimination and social/demographic covariates)	1.08 (0.76-1.54)
Racial group (adjusted for social/demographic, behavioral, and medical covariates)	1.17 (0.85-1.63)
