# Supplementary material for: Worry about racial discrimination: A missing piece of the puzzle of Black-White disparities in preterm birth?
Source: PLoS One. 2017 Oct 11;12(10):e0186151. doi: 10.1371/journal.pone.0186151 (PMC5636124; doi:10.1371/journal.pone.0186151)
Supplement: S7 Table — (PDF) [file pone.0186151.s007.pdf]

S7 Table. P-values from chi-square tests for association between maternal stressors and preterm birth, U.S.-born non-Latino Black and White women with singleton live births in California, MIHA 2011-2014.

	U.S.-born Black women (n=2,201)	U.S.-born White women (n=8,122)
Separation or divorce during pregnancy	0.576	0.116
Had to move due to difficulty paying rent or mortgage	0.803	0.995
Homeless or housing insecure	0.207	0.020
Respondent or partner lost job	0.730	0.866
Respondent or partner had pay or hours cut	0.345	0.581
Respondent or partner went to jail	0.076	0.753
Someone close to respondent had a serious drug or alcohol problem	0.687	0.528
Intimate partner violence during pregnancy	0.668	0.412
Food insecurity during pregnancy	0.331	0.019
