# Supplementary material for: Worry about racial discrimination: A missing piece of the puzzle of Black-White disparities in preterm birth?
Source: PLoS One. 2017 Oct 11;12(10):e0186151. doi: 10.1371/journal.pone.0186151 (PMC5636124; doi:10.1371/journal.pone.0186151)
Supplement: S8 Table — (PDF) [file pone.0186151.s008.pdf]

S8 Table. Rates of preterm birth and chronic worry about racial discrimination, according to detailed maternal race and partner race, among U.S.-born non-Latino White women with singleton live births in California, MIHA 2011-2014.

Among U.S.-born White women in the sample:	Percentage (N)	Preterm birth rate	Percent reporting chronic worry about discrimination
Maternal race			
Reported White only	96.7 (n=7,698)	5.8 (4.8-6.8)	5.3 (4.3-6.3)
Reported White (first race) and Black as second or third race	0.6 (n=71)	3.7 (0.0-8.1)	20.1 (8.5-31.7)
Reported White (as first race) and another racial/ethnic group(s) as second or third race	2.7 (n=353)	5.1 (2.4-7.8)	8.6 (4.6-12.6)
Paternal race			
White only, non-Latino	73.1 (n=5,886)	5.6 (4.4-6.8)	4.8 (3.6-5.9)
Black (1st, 2nd, or 3rd race) (includes Latino and non-Latino fathers)	2.8 (n=281)	4.7 (1.8-7.6)	15.0 (6.9-23.2)
Other racial/ethnic group(s) or missing paternal race/ethnicity	24.1 (n=1,955)	6.5 (4.7-8.3)	6.6 (4.6-8.6)
